# Sequences of Regressions Distinguish Nonmechanical from Mechanical Associations between Metabolic Factors, Body Composition, and Bone in Healthy Postmenopausal Women^1^,^2^,^3^

**DOI:** 10.3945/jn.115.224485

**Published:** 2016-03-09

**Authors:** Ivonne Solis-Trapala, Inez Schoenmakers, Gail R Goldberg, Ann Prentice, Kate A Ward

**Affiliations:** 4 Nutrition Studies and Surveys and; 5 Nutrition and Bone Health, Medical Research Council Human Nutrition Research, Elsie Widdowson Laboratory, Cambridge, United Kingdom; and; 6 Health Services Research Unit, Institute for Science and Technology, Keele University, Staffordshire, United Kingdom

**Keywords:** peripheral quantitative computed tomography, bone, adiponectin, leptin, osteocalcin, graphical Markov model, postmenopausal women, fat-free mass, fat mass, BMI

## Abstract

**Background:** There is increasing recognition of complex interrelations between the endocrine functions of bone and fat tissues or organs.

**Objective:** The objective was to describe nonmechanical and mechanical links between metabolic factors, body composition, and bone with the use of graphical Markov models.

**Methods:** Seventy postmenopausal women with a mean ± SD age of 62.3 ± 3.7 y and body mass index (in kg/m^2^) of 24.9 ± 3.8 were recruited. Bone outcomes were peripheral quantitative computed tomography measures of the distal and diaphyseal tibia, cross-sectional area (CSA), volumetric bone mineral density (vBMD), and cortical CSA. Biomarkers of osteoblast and adipocyte function were plasma concentrations of leptin, adiponectin, osteocalcin, undercarboxylated osteocalcin (UCOC), and phylloquinone. Body composition measurements were lean and percent fat mass, which were derived with the use of a 4-compartment model. Sequences of Regressions, a subclass of graphical Markov models, were used to describe the direct (nonmechanical) and indirect (mechanical) interrelations between metabolic factors and bone by simultaneously modeling multiple bone outcomes and their relation with biomarker outcomes with lean mass, percent fat mass, and height as intermediate explanatory variables.

**Results:** The graphical Markov models showed both direct and indirect associations linking plasma leptin and adiponectin concentrations with CSA and vBMD. At the distal tibia, lean mass, height, and adiponectin-UCOC interaction were directly explanatory of CSA (*R*^2^ = 0.45); at the diaphysis, lean mass, percent fat mass, leptin, osteocalcin, and age-adiponectin interaction were directly explanatory of CSA (*R*^2^ = 0.49). The regression models exploring direct associations for vBMD were much weaker, with *R*^2^ = 0.15 and 0.18 at the distal and diaphyseal sites, respectively. Lean mass and UCOC were associated, and the global Markov property of the graph indicated that this association was explained by osteocalcin.

**Conclusions:** This study, to our knowledge, offers a novel approach to the description of the complex physiological interrelations between adiponectin, leptin, and osteocalcin and the musculoskeletal system. There may be benefits to jointly targeting both systems to improve bone health.

## Introduction

The relation between bone metabolism and fat tissue/adipocyte-derived adipokines is not well understood, nor are any resultant effects on volumetric bone mineral density (vBMD)^7^, bone size [cross-sectional area (CSA)], distribution (e.g., cortical area, cortical thickness), and estimates of strength (e.g., stress strain index). Evidence suggests a complex interaction between metabolic factors (e.g., leptin, adiponectin, osteocalcin) and mechanical loading through muscle force and body weight (1, 2).

Leptin is secreted by adipocytes, and its plasma concentration is positively associated with fat mass; in turn, both are positively associated with bone mineral density (BMD). Leptin also acts directly on osteoblasts by upregulating bone formation (3). In contrast, there is evidence of negative effects of leptin on bone metabolism via the central nervous system mechanisms regulating body weight (4–6). Both positive and negative associations have been reported between leptin and BMD (7–12).

Adiponectin is also secreted by adipocytes, and the regulation of secretion is thought to be primarily related to adipocyte size and insulin sensitivity (13). Greater plasma concentration of adiponectin is associated with better insulin sensitivity, lower fat mass and inflammation, and greater lean mass and muscle strength (14–16). Better insulin sensitivity, in turn, indirectly stimulates osteoblast activity (2, 4, 17–19). Adiponectin receptors are also found on both osteoblasts and osteoclasts, suggesting direct effects on bone turnover (20–22). In human studies, negative associations of adiponectin with fat mass, BMD, and cortical thickness have been reported, and adiponectin has been shown to be a predictor of fracture in men (14, 23–30).

Osteocalcin is a bone matrix protein synthesized in the osteoblast. Osteocalcin is formed when preosteocalcin undergoes posttranslational modification, which includes vitamin K–dependent (phylloquinone or menaquinone) carboxylation (31). The plasma concentration of osteocalcin is a marker of bone formation. As a fraction of osteocalcin and undercarboxylated osteocalcin (UCOC) is incorporated into the bone matrix, the plasma concentration of both may also be influenced by the rate of bone resorption (19). An increased ratio of circulating UCOC to osteocalcin has been linked to poor bone health (32). UCOC may also have an endocrine function and has also been reported to stimulate adiponectin secretion by adipocytes, but evidence is inconsistent (18, 19, 33, 34).

Considering the interlinked nature of bone metabolism, fat and energy metabolism, and body composition, it is complex but relevant to distinguish between the direct actions of leptin, adiponectin, and osteocalcin on bone and their indirect actions via body composition (4, 14, 35). Sequences of Regressions (36, 37), a subclass of graphical Markov models (38, 39), is a multivariate statistical technique that extends path analysis and provides, to our knowledge, a novel strategy for describing complex interrelations by jointly modeling multiple outcomes and background factors (40). We hypothesized that there would be nonmechanical associations of the metabolic factors with bone size, distribution, and vBMD in addition to the mechanical effects of loading via fat, lean mass, and height. The primary aim of this study was to use Sequences of Regressions to simultaneously describe the interrelations between metabolic factors (leptin, adiponectin, and osteocalcin) and bone CSA, cortical area, and vBMD, measured by peripheral quantitative computed tomography (pQCT), and whether these were direct through metabolic pathways or indirect (i.e., mechanical) through body composition or height. The secondary aim was to investigate whether specific associations between metabolic factors, bone, and body composition were supported by the graphical Markov model.

## Methods

### Participants

Seventy white British postmenopausal women residing in Cambridge, United Kingdom, were selected by the use of a stratified design by age and BMI (in kg/m^2^) as defined in the inclusion criteria (41). The original cross-sectional study was powered to detect a correlation of 0.3 between adiponectin and BMD at 90% power and 0.05 significance level (41). Recruitment was through the Medical Research Council Human Nutrition Research volunteer database and advertisement in general practitioner clinics, media, and local clubs and educational establishments. Inclusion criteria were aged between 55 and 70 y, had last menstruated >6 y before recruitment, and BMI between 18.5 and 35.0. Exclusion criteria were early menopause (<45 y); had either a hip replacement or other metal implant; had been diagnosed with osteoporosis, diabetes, or a thyroid condition; or were taking any of the following medications known to affect BMD: steroids, statins, cholestyramine, and antacids containing aluminum. Ethical approval was obtained from the Cambridge Local Research Ethics Committee. All participants gave written informed consent. The study was carried out in accordance with the Declaration of Helsinki.

### pQCT

A Stratec XCT-2000 (Stratec Medizintechnik, Pforzheim, Germany) pQCT scanner was used. Scans of the nondominant tibia and nondominant radius were taken at 2 sites: *1*) tibia 4% (distal) and 38% (diaphysis) and *2*) radius 4% (distal) and 33% (diaphysis) (42). At the distal site, measurements of total and trabecular vBMD (mg/cm^3^) and total cross-sectional area (mm^2^) [cross-sectional bone area of the distal site (CSA-dis)] were taken. The diaphysis measurements were cortical vBMD (mg/cm^3^), cortical area (mm^2^), total CSA [cross-sectional bone area of the diaphysis site (CSA-dia)], and stress strain index (SSI) (mm^2^). To define the scan sites, a scout view was taken and the reference line was positioned through the middle of the distal endplate. Scans were analyzed with the use of the following parameters: distal site—contour mode 1, peel mode 1, threshold of 180 mg/cm^3^ and diaphyseal site—separation mode 1, threshold 710 mg/cm^3^. Precision (CV%) of duplicate measurements of the tibia in our center (*n* = 30 adults) was 1.1% and 1.6% (distal) and 0.4% and 1.0% (diaphysis) for vBMD and CSA, respectively. Repeat radius measurement CVs ranged between 1% and 4%.

### Assessment of body composition with the use of a 4-compartment model

Fat mass (FM, kg) was calculated by the use of the equations of Fuller et al. (43), which requires measurements of 4 compartments: total body water (L), total bone mineral content (kg), total body volume (L), and body weight (BW, kg). The body volume and BW (mass) variables function together within the model to provide a measure of density (mass/volume), which then takes into account the water and mineral content of the body represented by the other variables. This leaves the fat and protein compartments to be separated based on assumptions about their density. In this study, total body water was assessed by deuterium dilution with the use of isotope ratio MS, bone mineral content by DXA (Lunar MD machine; GE Lunar Corp.), and body volume and BW were calculated by air-displacement plethysmography (Bod Pod; Life Measurement Instruments). FM percent [(FM/BW) × 100] was used as a measure of adiposity. Fat-free mass was calculated by subtracting FM from body weight and was an estimate of lean mass.

### Biochemistry

A fasting, early morning blood sample was taken on the same day as bone and body composition measurements, and lithium heparin plasma was stored at −80°C. Plasma total adiponectin (Alpco diagnostics) and leptin (R&D Systems Europe, Ltd.) concentrations were measured by the use of sandwich ELISAs. Plasma osteocalcin (1–43) and UCOC concentrations were measured by automated sandwich chemiluminescence assay (Diasorin, Inc.) and radioimmunoassay (UCOC; Takara, Shuzo Co. Ltd.), respectively. [We were unable to calculate percent UCOC from the 2 assays used in our analysis because of differences in the standardization and sensitivity to circulating fragments (44).] Phylloquinone was measured by HPLC (Waters 2790/2690; Waters UK) with fluorescence detection (Waters 474; Waters UK) (45). All biochemical measurements were performed in duplicate. The intra-assay CVs for duplicate measurements were 0–5.4% for adiponectin, 3.3% for leptin, 1.9–11.9% for osteocalcin, 0.2–9.5% for UCOC, and 3.0–8.2% for phylloquinone. The interassay CVs were 8.6–10.2% for adiponectin, 4.4% for leptin, 4.0–9.8% for osteocalcin, 1.4–7.0% for UCOC, and 8.1–16% for phylloquinone.

### Anthropometric measurements

Height was measured in bare feet, to the nearest 0.1 cm, by the use of a calibrated portable stadiometer (CMS Weighing Equipment Ltd.). Weight was measured (Bod Pod) to the nearest 0.01 kg while the subject was in a fasting voided state and wearing a swimsuit, with no jewelry, watches, or glasses.

### Statistical methods

#### Primary analysis.

**FIGURE 1:**
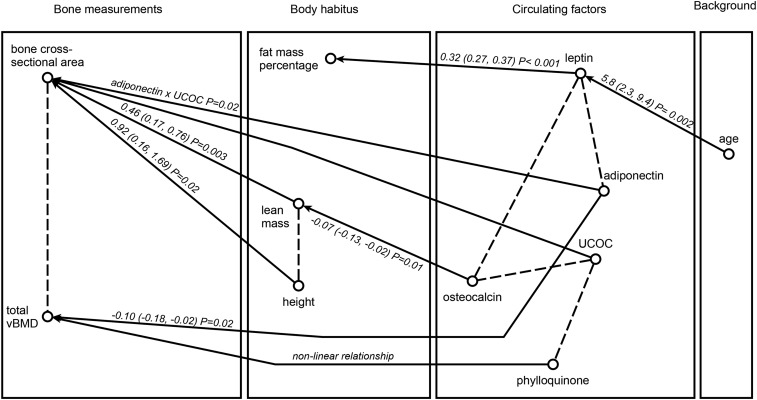
Fitted regression graph for distal tibia phenotype, body habitus, and biochemical markers of bone and fat, based on data from 70 postmenopausal women. In the regression graph, an arrow is present between a response and an explanatory variable if there is a significant association, controlling for all its remaining regressors. The strength of this association is shown as a partial regression coefficient (95% CI) and *P* value. Significant interactions described in Table 2 and Figure 3 and nonlinear relations described in Table 2 are also indicated. A dashed line connects 2 response variables within the same box if there is a significant association between them, controlling for their combined set of explanatory variables. UCOC, undercarboxylated osteocalcin; vBMD, volumetric bone mineral density.

We used Sequences of Regressions (36, 37), a graphical Markov model to build a multivariate model for sets of multiple response variables of bone phenotype (vBMD, CSA, and cortical area of the tibia), metabolic factors (leptin, osteocalcin, adiponectin, UCOC, phylloquinone), and body habitus (FM percentage, lean mass, height). The aim of these models was to statistically describe the hypothesized interrelations among multiple response variables in a single model to identify the relevant mechanistic links, rather than to determine the set of best predictors for each variable as would have been the case if traditional linear regression was used. The interrelations among variables were depicted by a regression graph with nodes representing variables arranged in blocks and connected by arrows or lines to represent direct and indirect associations, respectively (**Supplemental Methods**). To specify the model, based on our hypotheses, we defined an a priori order of the variables (**Figures 1** and ** 2**) in blocks of multiple-response variables and age (participant characteristic in the last box). The blocks of variables were ordered to simultaneously describe direct associations between bone and metabolic factors that were not explained by their relation with height, FM percentage, and lean mass. Such relations were assumed to be reflective of nonmechanical associations between metabolic factors and bone phenotype. Indirect associations (i.e., those via lean mass, FM, and height) were also described.

**FIGURE 2:**
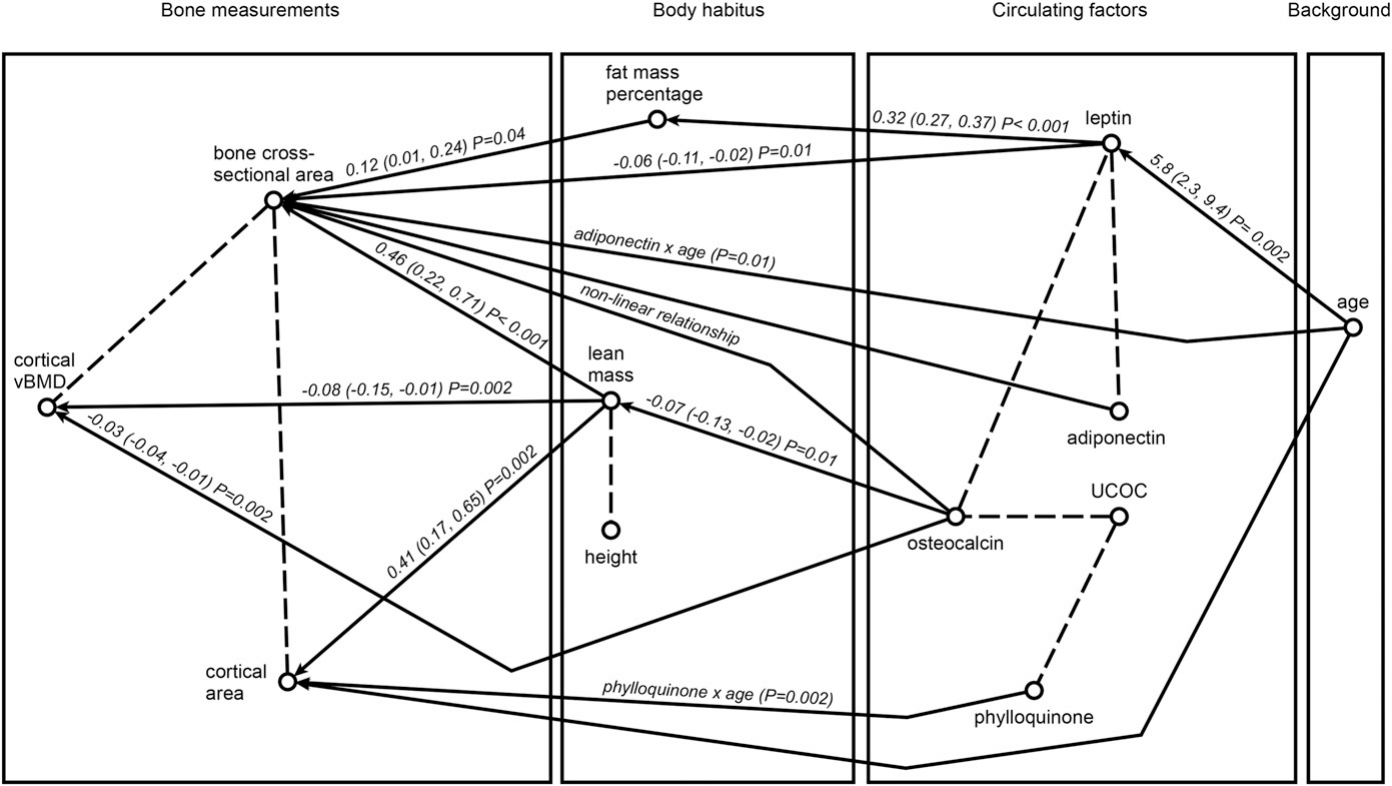
Fitted regression graph for diaphyseal tibia phenotype, body habitus, and circulating factors, based on data from 70 postmenopausal women. In the regression graph, an arrow is present between a response and an explanatory variable if there is a significant association, controlling for all its remaining regressors. The strength of this association is shown as a partial regression coefficient (95% CI) and *P* value. Significant interactions described in Table 2 and Figure 3 and nonlinear relations described in Table 2 are also indicated. A dashed line connects 2 response variables within the same box if there is a significant association between them, controlling for their combined set of explanatory variables. UCOC, undercarboxylated osteocalcin; vBMD, volumetric bone mineral density.

**FIGURE 3:**
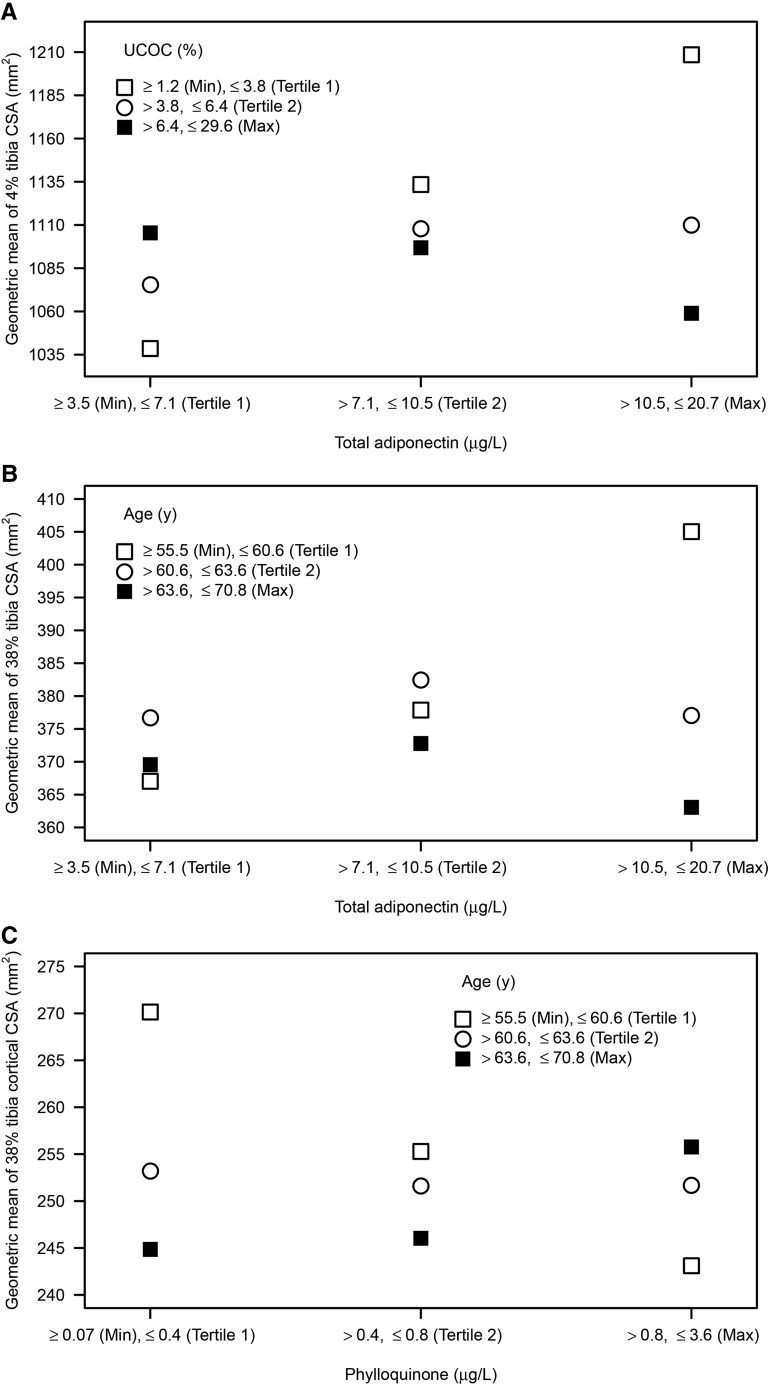
Plots of interactions identified in the graphical models, based on analysis of data from 70 postmenopausal women, for (A) the interaction of adiponectin plasma concentration and UCOC on distal tibia bone cross-sectional area, *P*-interaction = 0.02; (B) the interaction of adiponectin plasma concentration and age on diaphyseal bone cross-sectional area, *P*-interaction = 0.01; and (C) the interaction of phylloquinone and age on diaphyseal tibia bone cortical area, *P*-interaction = 0.002. The 3 points represent the bone phenotype geometric mean at different plasma concentrations of adiponectin and UCOC (A), age and adiponectin (B), and age and phylloquinone (C) (data split by tertiles of the observed distributions). Geometric means were used because the model estimated means of log-transformed data, which is equivalent to estimating geometric means in the original data. CSA, cross-sectional area; Max, maximum data value; Min, minimum data value; UCOC, undercarboxylated osteocalcin.

The above model was built by fitting ordered sequences of regression models for each variable in the blocks of multiple-response variables. A detailed description of model estimation and interpretation, including checks for interaction terms and nonlinear effects of explanatory variables, is provided in the Supplemental Methods. Partial regression coefficients from the linear least squares regressions were used to quantify the relative importance of associations depicted by the arrows in the graph. These could be positive or negative associations for a positive or negative sign of the partial regression coefficient, respectively. All variables were transformed to natural logarithms before analysis to allow interpretation of a partial regression coefficient as a percentage change in the response variable for a percentage change in the explanatory variable (46). The nonmechanical and mechanical associations are reported below as partial regression coefficients (β) and 95% CIs. For ease of interpretation, these associations can be read off the regression graphs from right (direct or intermediate explanatory variables) to left (response variables). To aid interpretation of 2-way interactions, we split the main factors into tertiles to represent low, medium, and high levels and plotted them against geometric mean values of the outcome (**Figure 3**). The level of statistical significance was set at 5%.

Although the model fitting required multiple statistical tests, the components of the model were carefully chosen to reflect distinctive relations of interest whose interpretation is of interest on their own. Therefore, the interpretation of each significance level reported is valid, and adjustment for multiple testing is not required (47).

#### Secondary analysis.

The regression models describing the association between any 2 outcome variables in the primary analysis partitioned out the effects of their combined sets of explanatory variables. The global Markov property of the fitted regression graphs (Figures 1 and  2) was used to further investigate the relation between 2 variables of interest after partitioning out the effects of variables that do not necessarily include their directly explanatory variables (Supplemental Methods) (37). The presence of a relation depends on how the 2 variables are linked through the other variables in the model. To identify the role of important variables more clearly, we explored whether the data supported associations between *1*) vBMD and plasma leptin and adiponectin concentrations after partitioning out the effect of only FM percentage and *2*) lean mass with UCOC after partitioning out the effect of osteocalcin.

#### Further analyses.

Tibia pQCT measures were the primary bone outcomes because the tibia is weight-bearing and measurements are the most precise. We replicated the analyses on measurements of the radius to assess the consistency of our findings. In addition, models were fitted with and without trabecular vBMD (4% site) and SSI (38% site) to select the most parsimonious models.

All analyses were performed with the use of the statistical software R (R Development Core Team). The “ggm” package in R provides tools for reading interrelations off the graph with the use of the global Markov property of the graph (48).

## Results

Descriptive statistics are given in **Table 1**, and **Table 2** presents a summary of the sequences of regressions fitted for separate response variables.

**TABLE 1:** Descriptive characteristics of the study population of United Kingdom postmenopausal women^1^

Variable	Values
Age, y	62.3 ± 3.7 (55.5–70.9)
Height, m	1.7 ± 0.1 (1.5–1.8)
Weight, kg	67.5 ± 11.3 (49.2–100.0)
BMI,^2^ kg/m^2^	24.9 ± 3.8 (17.6–33.3)
Lean mass, kg	47.6 ± 4.2 (38–57.5)
Fat mass, %	29.2 ± 8.1 (10.9–43.8)
Menopausal age, y	51.6 ± 3.9 (41.7–63.5)
Years since menopause, y	10.7 ± 5 (3.2–29.2)
Lumbar spine *T* score^3^	0.86 ± 1.2 (−4.0 to 2.2)
Femoral neck *T* score^4^	-1.0 ± 0.73 (−2.4 to 0.90)
4% tibia CSA, mm^2^	1110 ± 121 (866–1440)
4% tibia total BMD, mg/cm^3^	269 ± 35.2 (182–356)
38% tibia CSA, mm^2^	376 ± 37 (296–474)
38% tibia cortical CSA, mm^2^	253 ± 22.6 (203–301)
38% tibia cortical BMD, mg/cm^3^	1120 ± 26.7 (1050–1170)
Plasma total adiponectin, μg/L	9.4 ± 3.5 (3.5–20.7)
Plasma leptin, μg/L	18.5 ± 15.2 (1.0–87.0)
Plasma osteocalcin, μg/L	21.2 ± 7.8 (9.2–43.6)
Plasma UCOC, μg/L	7.4 ± 6.9 (1.2–29.6)
Plasma phylloquinone, μg/L	0.8 ± 0.7 (0.1–3.6)

1 Values are means ± SDs (ranges), *n* = 70. BMD, bone mineral density; CSA, cross-sectional area; UCOC, undercarboxylated osteocalcin.

2 Of the participants, 63% had a healthy BMI (in kg/m^2^; 18.5–24.9), 19% were overweight (BMI 25–29.9), and 16% were obese (BMI ≥30).

3 *n* = 68 from DXA (GE Lunar Prodigy) measurement of spine.

4 *n* = 69 from DXA (GE Lunar Prodigy) measurement of hip.

**TABLE 2:** Selected regression models from regression graphs describing direct and indirect associations between bone outcomes, circulating factors, and body composition in 70 postmenopausal women^1^

	Dependent variable				
	Distal tibia^2^		Diaphyseal tibia^3^		
Explanatory variables	Cross-sectional area (*n* = 61)	Total vBMD (*n* = 60)	Cross-sectional area (*n* = 60)	Cortical area (*n* = 62)	Cortical vBMD (*n* = 62)
Adiponectin	0.21 (0.07, 0.35)	−0.10 (−0.18, −0.02)	4.9 (1.1, 8.7)		
UCOC	0.87 (0.15, 1.6)				
Adiponectin × UCOC	−0.10 (−0.18, −0.02)				
Adiponectin × age			−1.2 (−2.09, −0.3)		
Phylloquinone		0.06 (0.01, 0.11)		−2.6 (−4.2, −1.1)	
Phylloquinone squared term		0.03 (0.001, 0.06)			
Phylloquinone × age				0.64 (0.26, 1.0)	
Lean mass	0.46 (0.17, 0.76)		0.46 (0.22, 0.71)	0.41 (0.17, 0.65)	−0.08 (−0.15, −0.01)
Height	0.92 (0.16, 1.7)				
Osteocalcin			−1.1 (−1.8, −0.3)		−0.03 (−0.04, −0.01)
Osteocalcin squared term			0.2 (0.06, 0.31)		
Age			11 (2.4, 19)	0.27 (−0.14, 0.68)	
Fat mass percentage			0.12 (0.01, 0.24)		
Leptin			−0.06 (−0.11, −0.02)		
*R*^2^	0.45	0.15	0.49	0.27	0.18

1 Partial regression coefficients, β (95% CI), and *R*^2^ from the Sequences of Regressions were used to estimate the graphical Markov model. Significance levels were set at *P* < 0.05. The regression coefficients can be interpreted as percent differences in the outcome per 1% difference in explanatory variable, holding the other variables fixed. For example, a 1% difference in lean mass would correspond to a 0.46% difference in bone cross-sectional area. UCOC, undercarboxylated osteocalcin; vBMD, volumetric bone mineral density.

2 Cross-sectional area and total vBMD were associated after controlling for their combined explanatory variables (results not shown).

3 Cross-sectional area was associated with vBMD and cortical area, but vBMD and cortical area were not associated after controlling for their combined set of explanatory variables (results not shown).

For the distal tibia, the variation in CSA-dis was explained by a combination of lean mass, height, and plasma concentrations of adiponectin and UCOC individually and in an interaction term (coefficient of determination *R*^2^ = 0.45). Variation in total vBMD was explained by adiponectin and phylloquinone concentrations (*R*^2^ = 0.15). At the tibia diaphysis (38% site), variation in CSA-dia was explained by lean mass, FM percentage, leptin concentration, osteocalcin concentration, and age and adiponectin, individually and in an interaction term (*R*^2^ = 0.49). Variation in cortical area was explained by lean mass and phylloquinone concentration and age, individually and in an interaction term (*R*^2^ = 0.27). Finally, variation in cortical vBMD was explained by lean mass and osteocalcin concentration (*R*^2^ = 0.18).

For body composition, variation in FM percentage was explained by leptin concentration (*R*^2^ = 0.71), whereas variation in lean mass was explained by osteocalcin concentration (*R*^2^ = 0.09). It is important to remember that relations between variables contained in the same box are not reported, which is why the well-known relation between height and lean mass is not described and why the *R*^2^ for lean mass is so low.

The interrelations among all the variables are described in Figure 1 (tibia 4%, distal) and Figure 2 (tibia 38%, diaphyseal).

### Nonmechanical associations.

There was a direct, negative association between leptin and CSA-dia. A 10% greater leptin concentration was associated with a 0.6% decrease in CSA (partial regression coefficient, β: −0.06%; 95% CI: −0.11, −0.02). This association was direct (i.e., not explained) by the relation between leptin and FM percentage.

There was a significant interaction term between plasma adiponectin concentration and UCOC in the regression model for CSA-dis. Although the individual partial regression coefficients are positive for both variables, the interaction term has a negative coefficient, indicating a reducing effect of UCOC on the size of the relation between adiponectin and CSA-dis. For those with the lowest concentration of UCOC, there was a positive relation between adiponectin and CSA and a negative, slightly weaker, relation for those with the highest concentration of UCOC (Figure 3A). There was a significant interaction term between plasma adiponectin concentration and age on CSA-dia, indicating a reducing effect of age on the size of the relation between adiponectin and CSA-dia. The estimated geometric mean of CSA-dia was 10% smaller for participants in the upper tertile of age (aged >64 y) who had a plasma adiponectin concentration >10.5 mg/L (the upper tertile of the observed distribution) than for the group of participants aged <61 y (lower tertile) with a similar concentration of adiponectin. No relation was evident for participants with a lower adiponectin concentration (Figure 3B).

Adiponectin concentration was directly, negatively associated with vBMD of the distal tibia, which was not explained by body composition (β: −0.10%; 95% CI: −0.18, −0.02). Furthermore, the global Markov property of the graph indicated a significant association of plasma concentrations of leptin with adiponectin and vBMD.

There were significant, direct associations between osteocalcin concentration and CSA-dia and cortical vBMD. There was a direct quadratic association between osteocalcin concentration and CSA-dia, where CSA-dia was greater in individuals with low and high osteocalcin concentrations. A 10% greater concentration of osteocalcin was associated with a 0.3% lower cortical vBMD (β: −0.03; 95% CI: −0.04, −0.01).

There was a significant interaction between phylloquinone status and age for cortical area at the diaphyseal tibia. In women with poorer phylloquinone status, younger participants had a greater cortical area (i.e., thicker cortices) than older participants (Figure 3C). In contrast, better phylloquinone status was associated with a 3% greater cortical area in older participants.

Osteocalcin concentration was directly, negatively associated with lean mass (β: −0.07; 95% CI: −0.13, −0.02). With the use of the global Markov property of the graph, the data show that while lean mass and UCOC were related, their relation was explained by osteocalcin, because the association disappeared when the effect of osteocalcin was partitioned out.

Leptin concentration was directly, positively associated with FM percentage (β: 0.32; 95% CI: 0.27, 0.37).

### Mechanical associations.

Lean mass was directly, positively associated with CSA-dia (β: 0.46; 95% CI: 0.17, 0.76), CSA-dis (β: 0.46; 95% CI: 0.22, 0.71), and cortical area (β: 0.41; 95% CI: 0.17, 0.65) and was directly, negatively associated with cortical vBMD (β: −0.08; 95% CI: −0.15, −0.01). FM percentage was directly, positively associated with CSA-dia (β: 0.12; 95% CI: 0.01, 0.24) but not CSA-dis. Height was directly, positively associated with CSA-dis (β: 0.92; 95% CI: 0.16, 1.7) and was indirectly associated with vBMD, CSA-dia, and cortical area through lean mass for the diaphyseal tibia.

### Consistency of results in further analyses.

Our findings reflecting the impact of nonmechanical factors on bone in the tibia were largely replicated in the radius, a non-weight-bearing site indicating the consistency of our results (see **Supplemental Table 1**, **Supplemental Figures 1** and **2**).

Inclusion of trabecular vBMD in the graphical Markov model for the distal tibia (results not shown) made no difference to interpretation; associations were consistent with those for total vBMD. Similarly, inclusion of SSI did not change the interpretation of results for the diaphyseal site; the same patterns of associations were found for SSI as with CSA-dia.

## Discussion

We have used, to our knowledge, a novel method of analysis to describe interrelations in postmenopausal women between leptin, adiponectin, osteocalcin, and bone after partitioning out the contribution of body composition. We reported evidence of direct, negative, and indirect positive, associations between leptin and CSA. Furthermore, we showed evidence that suggests a synergy between plasma concentration of adiponectin and osteocalcin. We also described relations between osteocalcin concentration and lean mass.

Greater percentage FM and higher leptin concentration were positively associated with CSA-dia, indicating the effect of greater adiposity on bone CSA. The direct negative association between leptin and bone CSA-dia may indicate the negative effects of leptin on the skeleton via central nervous system regulation of body weight, which have been demonstrated in mouse models but not consistently in humans (4–6, 10). One reason for inconsistencies in the literature may be limitations of data analyses, which may not have provided information on how the interrelation between FM and leptin affects the relation with CSA (4). Our modeling approach enabled the identification of partial direct and indirect relations between leptin and CSA-dia; the indirect relation was via FM.

The graphs for both the distal and diaphyseal tibia show significant associations that remain between leptin and adiponectin and vBMD after partitioning out the effect of FM percentage. These results support a potential nonmechanical role of adipose tissue on vBMD through adipocyte secretion of adiponectin and leptin and are consistent with previous reports (7–12, 14, 23, 24, 26, 27).

Our analyses showed a significant interaction between adiponectin and UCOC (distal tibia) or age (diaphyseal tibia). Individuals with higher adiponectin and either lower UCOC or younger age had greater bone CSA (Figure 3). Other studies have shown that higher plasma adiponectin concentrations are associated with better insulin sensitivity, lower FM and inflammation, and greater lean mass and muscle strength, all of which contribute to a greater bone CSA (14–16, 49). In contrast, for participants in the lowest tertile, there is lower bone loading due to low BMI (indicated by high adiponectin or older age) and consequently a smaller bone CSA. This phenotype is consistent with studies reporting positive associations between UCOC or adiponectin and increased risk of fracture because bones that are narrower in cross section for a given height are less strong (50).

Osteocalcin was directly, negatively associated with lean mass. The global Markov property of the graph demonstrated the consistency of this finding where an association between UCOC and lean mass existed only in the presence of osteocalcin in the model. This observation is consistent with previous findings showing an inverse relation between osteocalcin and BMI (15).

The predominant directly explanatory variable in the regression models for bone outcomes was lean mass. Following the mechanostat theory (51), greater lean mass and, consequently, body weight would exert greater force on bones, which would drive increases in bone CSA and cortical CSA to maintain strength. The negative association between lean mass and cortical vBMD may be because the cortex is thinner because the bone is bigger and longer (height was associated with cortical vBMD via lean mass) but needs to remain light while maintaining strength (52). Height was directly associated with CSA-dis and not total vBMD, indicating the allometric scaling of bone where a longer bone requires greater cross section to maintain strength. The lack of association with distal vBMD supports the notion that vBMD is governed by factors other than longitudinal growth (53, 54).

Our findings were largely replicated in the radius (Supplemental Table 1, Supplemental Figures 1 and 2). There was consistency between the tibia and radius for both nonmechanical and mechanical associations. Of note is the presence of interactions between UCOC and adiponectin at the radius. In addition, the nonmechanical associations found in the primary analysis were replicated in a model substituting lean mass with appendicular skeletal mass estimated by DXA (data not shown).

There are several strengths to this study. The use of independent methods (pQCT and Bod Pod) to measure outcomes and explanatory variables gives confidence that associations found would not be affected by the technical limitations of DXA (4, 43). DXA measurements of lean tissue, bone, and fat are determined from a single scan and are based on assumptions of the proportions of fat and lean mass in the soft tissue overlying bone, leading to an interdependence of measurements. We selected outcomes to provide a combined description of phenotype focusing on bone size, distribution, and vBMD. Although the population sample was small, it was carefully selected to represent a wide range of BMIs from underweight to obese, which increases the precision of the model parameter estimates. The analysis method used was designed to describe interrelations between bone, body composition, and metabolic factors in one multivariate statistical model, rather than describing predictive models for single outcome variables where confounding between explanatory variables can hamper the interpretation of study results. Furthermore, the model estimation is straightforward and transparent because it relies on the estimation of local regression analyses. This allows an efficient use of the data, provides a better insight into the interrelations, and allows the identification of nonlinear relations and checks of model assumptions. In contrast, structural equation models (55), which is an alternative approach widely used for generating and exploring new research hypotheses, can become increasingly complex as the number of parameters grows, leading to convergence problems and inadmissible solutions (56). Finally, our findings in the tibia were mostly replicated in the radius (Supplemental Methods), indicating the consistency of our results.

There are limitations to this study: *1*) The study design was cross-sectional and causality cannot be inferred; larger studies are required to test the consistency of the relations and implications for bone health in later life; *2*) Graphical Markov modeling aims to assess whether the data are consistent with postulated hypotheses reflected by the initial ordering of the variables. As with any method of analysis, it depends on having considered the most influential biological explanatory variables at the outset. In this study, we did not have detailed information of diet or physical activity to include in the model; *3*) We do not know whether our results would be confirmed with intact (1–49) osteocalcin; the osteocalcin assay we used measures the 1–43 osteocalcin molecule, and whether this is an active fragment or a breakdown product of the larger molecule is currently unknown (57); *4*) There may have been inaccuracy in the cortical vBMD measurement due to partial volume averaging due to thin cortices, but measurements were taken at sites where this should be minimal (58); and *5*) We did not have measures of muscle strength to substantiate our findings with fat-free mass.

In conclusion, our data support previous work showing the importance of a good metabolic health status and nutrient intakes for bone health and that there is a need to consider interactions between bone, fat, and muscle to understand healthy aging. This article provides a strategy for identifying these pathways simultaneously, as well as evidence to further inform future study and intervention designs that may target together musculoskeletal and metabolic health.

## Footnotes

7 Abbreviations used: BMD, bone mineral density; BW, body weight; CSA, cross-sectional area; CSA-dia, cross-sectional bone area of the diaphysis site; CSA-dis, cross-sectional bone area of the distal site; FM, fat mass; pQCT, peripheral quantitative computed tomography; SSI, stress strain index; UCOC, undercarboxylated osteocalcin; vBMD, volumetric bone mineral density.
